# Good personality and social well-being: The roles of orientation to happiness

**DOI:** 10.3389/fpubh.2023.1105187

**Published:** 2023-04-05

**Authors:** Xiaodan Xu, Yang Liu, Liying Jiao, Yongming Wang, Mengke Yu, Yidie Lai, Yingjun Zhang, Yan Xu

**Affiliations:** ^1^Beijing Key Laboratory of Applied Experimental Psychology, National Demonstration Center for Experimental Psychology Education, Faculty of Psychology, Beijing Normal University, Beijing, China; ^2^Department of Psychology, School of Education, Shanghai Normal University, Shanghai, China; ^3^School of Biology and Basic Medical Sciences, Medical College of Soochow University, Suzhou, China; ^4^Psychological Education and Counselling Center, Beijing Normal University, Beijing, China

**Keywords:** good personality, social well-being, orientation to meaning, orientation to pleasure, multiple mediation

## Abstract

**Introduction:**

Positive personality traits have been associated with personal well-being in previous research. However, the pathways through which positive personality may affect social well-being remain unclear. The present study hypothesized that the cognitive strategies for achieving well-being (i.e., orientation to happiness) mediate the association between good personality and social well-being in the Chinese culture.

**Methods:**

A survey including the Good Personality Questionnaire, Social Well-being Scales, and Orientations to Happiness was administered to 1,503 Chinese secondary school students and adults.

**Results:**

The results indicated that orientation to meaning mediated the relation between good personality and social well-being, but not orientation to pleasure.

**Discussion:**

This is in line with the normative well-being model and the cognition instrumental model of well-being, which contributes to developing more targeted interventions to promote social well-being in the Chinese cultural.

## 1. Introduction

Well-being is a complex construct that is crucial for psychological and physical health (1). In the field of positive psychology, it was generally defined as an optimal psychological functioning (2–4). Research on well-being converged in three domains: subjective well-being (i.e., positive emotional functioning) (5), psychological well-being (i.e., happiness that comes from realizing one’s potential) (6), and social well-being (i.e., examines people’s well-being in a social-ecological context) (7, 8). Social well-being distinguished itself from the other two types of well-being since it focuses on assessing people’s well-being in a macro social context rather than from an individual perspective (9, 10). Compared with the other two types of well-being, social well-being is relatively less studied. As was mentioned before, social well-being is critical for health (1, 11), and exploring its contributors would provide new sights to promote a healthy life.

Positive personality traits, such as extraversion, were among the most studied variables related to social well-being (9, 12–15). The fact that most contemporary personality theories rely heavily on western cultural, sociological, and philosophical assumptions about people (16) made it challenging for psychologists to comprehend how personality models may vary between cultures. Psychologists used to compare distinctions between people living in various parts of the world using Western theories, concepts, and instruments, which means people are assessed by using the standards that are potentially culturally unsuitable for them. According to the cultural-psychology perspective (16), the meanings and behaviors of a specific sociocultural context determine individuals’ personalities. To describe the variations among Chinese people in terms of their daily lives, a Chinese personality idea is applied in this essay.

As far as Chinese Confucianism is concerned, a good personality is a positive moral character. There is no doubt that Confucianism’s classics have had the most impact on Chinese culture than any other literary or intellectual work. The Confucian philosophers, typified by Confucius and Mencius, who proclaimed the idea of “benevolence” and championed “benevolent administration” highly valued goodness. The essential cultural and psychological underpinnings of “benevolence” in China were based on the fundamental intellectual presuppositions of Confucius, Mencius, and Confucian scholar Seosso regarding human nature’s intrinsic goodness and evil. The Chinese people’s collective memory has been shaped by this cultural essence of goodness, which has left its stamp on their national identity, national character, and essential aspects of their personality (17). Personality psychology study with Chinese cultural components focuses on the good personality. Therefore, studying good personality is a good starting point for investigating the unique characteristics of Chinese people. In sum, the present study aimed to examine how Confucianism’s good personality may impact the social well-being of Chinese people.

The Social-Cognitive Model of Normative Well-Being postulates that personality and cognition both play important roles in well-being. It underlines how personality may influence well-being via cognitive factors (18). The model also describes how cognitive factors interact to preserve well-being under normal conditions according to empirical study (19, 20). Therefore, this study aimed to explore whether good personality in the Chinese cultural context augmented cognitive resources (e.g., orientation to happiness) and then improved social well-being.

### 1.1. Good personality and social well-being

Social well-being refers to an assessment of a person’s circumstances and social roles (7). It consists of five fundamental elements, including the perception and evaluation of social integration (i.e., a sense of belonging to the society), social acceptance (i.e., human nature and society are viewed positively), social actualization (i.e., a belief that the society will evolve), social contribution (i.e., being aware of one’s social value), and social coherence (i.e., a sense of meaning living in the social world) (7, 13). The correlation between social well-being and (subjective and psychological) personal well-being is moderate (7, 21), suggesting that social well-being and personal well-being are related, but are different constructs (22). Social well-being deserves as much attention as personal well-being that is more often studied in the past. Given the naming of social well-being, it is influenced by the cultural context by nature. Specifically, western culture stresses individual autonomy, whereas individuals in eastern cultures, such as Chinese, value social embeddedness and care more about contributing to others, the country, and the society (8, 23).

Good personality is characterized by positive moral personality in Chinese Confucianism, referring to whether s/he will be helpful to others (24, 25). Structure, inclination, sociality, and morality are the fundamental characteristics of good personality (24, 25). In this sense, the four qualities of a good personality are integrity, altruism, amiability, and magnanimity (24, 25). Dispositions allude to the reality that people of various good personalities often express their words and acts with a specific moral slant. Dual processing systems theory states that people have two processing systems: deliberate processing (controlled processing), which involves slow, energy-intensive conscious processing; and intuitive processing (heuristic processing), which concerns quick, low-energy processing that allows people to act by their inclinations and inner thoughts (26). Kindness individuals behave in a beneficial way intuitively (26). A good personality is formed due to many different social influences, and personality also affects how other people perceive and react to you in social situations.

According to Hillson (1999), personality qualities can be classified as positive and negative (27). In terms of stress-coping, positive personality is associated with problem-focused coping strategies (e.g., positive reappraisal strategies) (28), whereas negative personality is related to emotional strategy [e.g., denial, venting of emotions (29, 30)]. This may partially explain why positive and negative personalities affect physical and mental health differently. Specifically, positive personality (e.g., openness, extraversion, agreeableness, and conscientiousness) is beneficial to mental health (31, 32), and it plays a significant role in promoting social well-being (9, 12, 15). In addition, the neural basis of the association between good personality and social well-being has also been revealed, which include the dorsolateral prefrontal cortex (13), the orbitofrontal cortex (14), the right orbitofrontal sulcus (33), and the left postcentral sulcus (33). In contrast, negative personality may be detrimental to mental health, with research showing that Machiavellianism and psychopathy made it challenging to achieve long-term happiness (34).

Though previous research showed that moral character shaped subjective well-being (35) and predicted life satisfaction (36), little is known about the influence of good personality on social well-being. Concerning social well-being, amongst few studies conducted in Chinese, one found its correlation with big five personality (37) and another with family harmony and social dedication (38). Good personality is rooted in the Chinese cultural context, and examining the contribution of good personality to social well-being can provide intervention studies with important targets to promote social well-being. Thus, it is worthwhile to investigate how good personality affects social well-being, and we assumed a positive relationship between them.

### 1.2. Orientation to happiness as potential mediator

The social-cognitive model of normative well-being emphasizes the importance of personality and cognitive abilities in well-being, and it also assumes that cognitive variables could mediate the well-being effects of personality (18). Many studies have validated the normative well-being model outside Chinese culture (19, 20). The present study investigated whether good personality contributes to social well-being via the cognitive resources associated with orientations to happiness. Some research showed that cognitive variables were instrumental to understanding the link between personality traits and well-being, such that personality traits influence how individuals choose their situations and experience life, which further affects their subjective well-being (39, 40). It was found that cognitive mechanisms partially contribute to subjective well-being by mediating personality traits, supporting the instrumental model (41).

The present study aimed to understand the association between good personality and social well-being by considering the potential mediating role of the cognitive strategies individuals use to seek social well-being (i.e., orientation to happiness). As aforementioned, good personality is positive moral personality with Chinese cultural components (24, 25). Thus, this paper provides an opportunity to explore cognitive mediating variables in relation to culture. Culture has been suggested to significantly influence people’ happiness orientations (42). In the Chinese culture, harmonious interpersonal relationships (43) and dedication to society (i.e., putting others’ needs before one’s own) (44) are highly valued. A study showed that contribution to society provided Chinese happiness (45). That said, Chinese emphasize the importance of pursuing happiness through orientation to meaning. Meanwhile, due to China’s rapid economic growth and increasing globalization, people are more likely to be exposed to commercial advertisements through mass media (46, 47). Chronic exposure to such information made some Chinese endorse materialistic values and believe wealth acquisition is the foundation of happiness (48, 49). Moreover, materialistic values may facilitate hedonism and pleasure-seeking (50). Taking these into account, orientation to meaning and orientation to pleasure are two important mediators to consider when studying good personality and social well-being, especially in Chinese culture. In addition, a study showed that manipulating happiness orientation to meaningful rather than pleasant experience promotes well-being, further supporting the potential mediating role of orientation to happiness (51).

Based on previous research, there are two distinct but complementary cognitive strategies that individuals can use to pursue well-being: Eudaimonia and Hedonia (52). Eudaimonia believes that well-being comes from fully utilizing and developing oneself (6, 45). However, from the hedonistic perspective, an individual can achieve well-being by engaging in pleasure (52, 53). In a similar vein, the Orientations to Happiness Scale developed by Peterson and colleagues (53) encompassed two subscales: orientation to meaning and orientation to pleasure, which corresponds to eudaimonia and hedonia, respectively.

Positive personalities were found to be associated with orientation to happiness. For instance, positive personalities such as extraversion, agreeableness, hope, curiosity, gratitude, bravery, and conscientiousness were found consistently positively related to orientation to meaning (54, 55). However, the results about orientation to pleasure was inconsistent. For example, extraversion, hope, bravery, fairness, creativity, honesty, prudence, and modesty were found positively (54, 55), while honesty-humility was found negatively associated with orientation to pleasure (55). Albeit this inconsistency, most positive personalities were found to be positively associated with orientation to pleasure. Accordingly, we propose that good personality would be positively associated with orientation to meaning and orientation to pleasure.

In addition, the relationship between orientation to happiness and well-being has been extensively examined in previous studies (56–60), and the findings were relatively consistent. Specifically, meaning orientation seemingly played a more important role than pleasure orientation in predicting subjective well-being (54, 56, 61). This can be explained as orientation to meaning leads to better emotional regulation (56, 62) and more attention paid to interpersonal relationships and the world around them (63), which in turn leads to more resources for constructing well-being. In contrast, orientation to pleasure does not lead to the development of long-lasting emotional resources, but only short-term emotional improvements (62). Notably, these studies mainly focused on the association between happiness orientation and *personal* well-being (i.e., subjective well-being and psychological well-being) (15, 52, 53, 57–60), leaving *social* well-being less investigated. There is only one study found that social well-being correlated positively with orientation to meaning, while its correlation with orientation to pleasure was nonsignificant (64). Given the limited evidence, more empirical studies are needed for results validation.

### 1.3. An overview of the current study

The present study aimed to examine the mechanisms underlying the association between good personality and social well-being by exploring the potential mediating role of orientation to happiness. It was hypothesized that good personality was positively related to social well-being, and orientation to happiness mediated this relationship.

## 2. Methods

### 2.1. Participants and procedure

Participants included high school, vocational college, and university students and those who had already graduated and started working. A total of 1,503 participants were included (age range: 14–57 years, *M*_age_ = 18.7 years, SD = 4.27, 543 males). Three participants did not report age (0.02%)，592 were between 14 and 16 years (39.39%), 317 were between 17 and 18 years (21.09%), 498 were between 19 and 25 years (33.13%), and 93 were over 25 years (6.19%). Five participants did not report subjective socioeconomic status, and the socioeconomic status ranged from 1 to 10 (*M* = 6.10, *SD* = 1.52).

The participants were informed that the survey would remain confidential and not be revealed to anyone else, so that they would be able to complete it honestly. Upon completing the informed consent, participants completed a survey that measured good personality, orientations to happiness, and social well-being either online or in the classroom.

### 2.2. Measures

#### 2.2.1. Good personality questionnaire

The Good Personality Questionnaire includes 15 items, covering four dimensions, namely integrity (4 items; e.g., “I can admit my mistakes to others truthfully”), altruism (5 items; e.g., “I will not hesitate to go to help when I see someone in danger”), amicability (3 items; e.g., “People around me think I’m an easy person to get close to”), and magnanimity (3 items; e.g., “I will help the people who have hurt me regardless past grievances”) (65). Participants were asked to rate their agreement or disagreement on a Likert scale from 1 (strongly disagree) to 5 (strongly agree). The reliability and validity of this questionnaire was proved to be satisfactory (24, 25, 66), and the Cronbach’s alpha coefficient of the whole questionnaire and the four subscales were 0.89, 0.79, 0.81, 0.82, and 0.73, respectively in the present study.

#### 2.2.2. Social well-being

The social well-being scale includes 15 items (7) containing five subscales, e.g., the actualization of society (3 items; e.g., “Society is constantly evolving and progressing”), the coherence of society (3 items; e.g., “It’s easy for me to understand what’s going on in the world”), the integration of society (3 items; e.g., “Being a part of my community makes me feel close to others”), the acceptance of society (3 items; e.g., “Other people’s problems matter to people”), and social contribution (3 items; e.g., “As part of my daily activities, I contribute to the community in any way I can”). Ratings are based on a 7-point Likert scale (1 = strongly disagree to 7 = strongly agree). The Cronbach’s alpha coefficient of the whole questionnaire and the five subscales were 0.89, 0.88, 0.76, 0.68, 0.93, and 0.75 in the present study.

#### 2.2.3. Orientations to happiness

The Orientation to Happiness scale has two 6-item subscales that measure meaning orientation (e.g., “It is my responsibility to improve the world”) and pleasure orientation (e.g., “It is always important for me to consider whether or not what I am doing will bring me joy before making a decision”) separately (53). Participants rated each item from 1 (extremely unlike me) to 5 (extremely like me). A higher subscale score implies a higher likelihood of endorsing that orientation. The Cronbach’s alpha coefficient in the present study were 0.79 and 0.73 for these two dimensions, which were satisfactory.

### 2.3. Statistical analysis

The analyses were performed in IBM SPSS 25 and Mplus 7. First, the correlations between the main variables were calculated. Then a multiple mediation analysis was conducted with Mplus 7 to examine the mediating role of the orientations to happiness between good personality and social well-being. Based on the nonparametric bootstrap method (5,000 samples), a 95% confidence interval (CI) containing no zero was considered significant (66).

## 3. Results

Table 1 presents correlations, means, and standard deviations between variables. Good personality was positively correlated with orientation to meaning (*r* = 0.49, *p* < 0.001), orientation to pleasure (*r* = 0.21, *p* < 0.001), and social well-being (*r* = 0.58, *p* < 0.001). Orientation to meaning was positively associated with orientation to pleasure (*r* = 0.38, *p* < 0.001) and social well-being (*r* = 0.62, *p* < 0.001). Orientation to pleasure (*r* = 0.32, *p* < 0.001) was positively correlated with social well-being (*r* = 0.32, *p* < 0.001).

**Table 1:** Analyses of descriptive statistics and correlations (*N* = 1,503).

	*M*	SD	1	2	3	4
Good personality	3.46	0.59	1			
Orientation to meaning	3.41	0.69	0.49^***^	1		
Orientation to pleasure	3.39	0.67	0.21^***^	0.38^***^	1	
Social well-being	4.71	0.89	0.58^***^	0.62^***^	0.32^***^	1

^***^p < 0.001.

Two latent variables (good personality, social well-being) and 9 observed variables formed the measurement model. A significant factor loading was observed for each indicator (*ps* < 0.001), which indicated that the observed indicators adequately reflected the two latent variables. The measurement model satisfactorily fitted the data: *χ^2^* = 296.69, df = 26, *p* < 0.001; RMSEA = 0.08; SRMR = 0.04; CFI = 0.94; TLI = 0.92.

The mediation model with good personality as the independent variable, orientation to meaning and orientation to pleasure as mediating variables, and social well-being as the dependent variable were examined. Since age was positively correlated with good personality(*r* = 0.10, *p* < 0.001), orientation to meaning (*r* = 0.08, *p* < 0.01), and social well-being (*r* = 0.10, *p* < 0.001), The mediation model was run will age controlled. As three participants lacked age information, the analyses involved 1,500 participants.

According to Table 2, good personality has a significant impact on social well-being (*β* = 0.41, *p* < 0.001) and orientation to meaning (*β* = 0.53, *p* < 0.001). Furthermore, orientation to meaning had a significant effect (*β* = 0.46, *p* < 0.001) on social well-being, as did not orientation to pleasure (*β* = 0.02, *p* = 0.41).

**Table 2:** Model SEM results.

Hypotheses			Estimate	S.E.	β
Good personality	→	Social well-being	0.36	0.05	0.41^***^
Good personality	→	Orientation to meaning	0.82	0.06	0.53^***^
Good personality	→	Orientation to pleasure	−9.3	4.29	−0.09
Orientation to meaning	→	Social well-being	0.27	0.03	0.46^***^
Orientation to pleasure	→	Social well-being	0.00	0.00	0.02

***p < 0.001.

**Figure 1:**
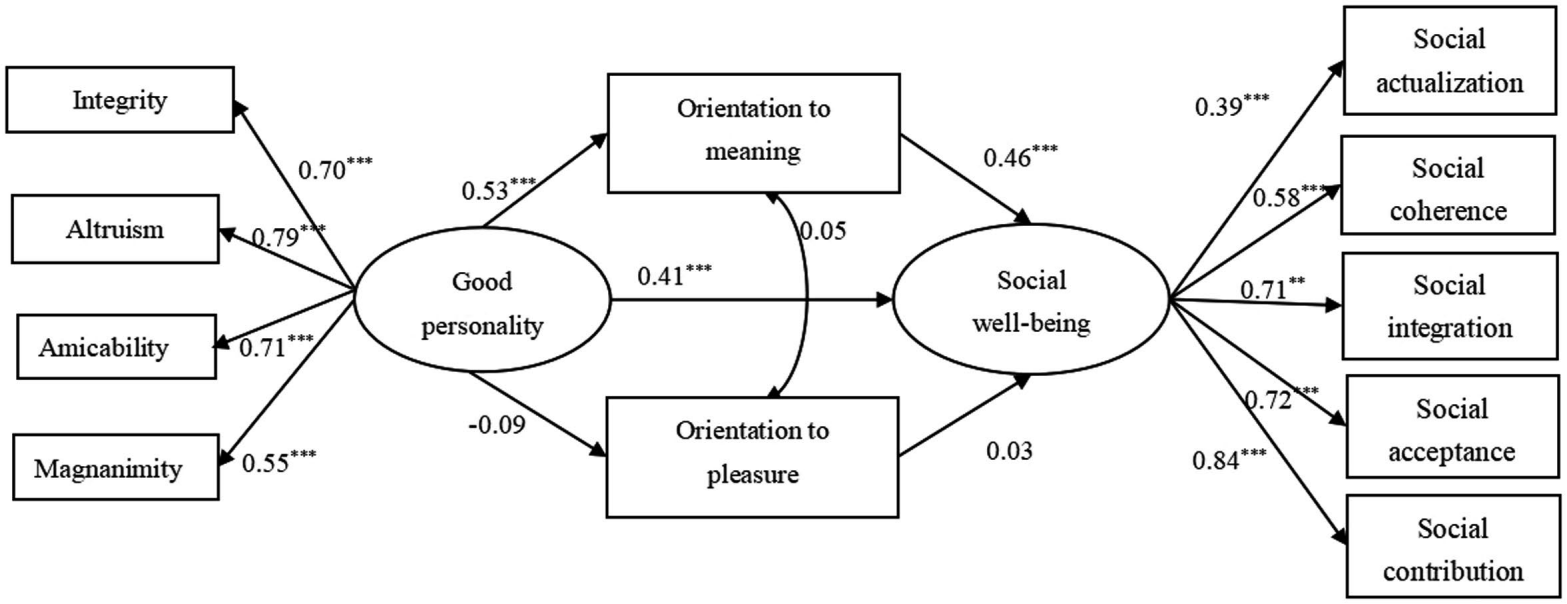
Shows the direct effects of the variables. Model fitted well to the data (*χ^2^* = 228.52, df = 44, *p* < 0.001; RMSEA = 0.08; SRMR = 0.04; CFI = 0.93; TLI = 0.90).

As shown in Table 3, orientation to meaning (95% CI = [0.20, 0.29]) partially mediated the association between good personality and social well-being, while the mediating effect of orientation to pleasure was not significant (95% CI = [−0.01, 0.003]), as was shown in Figure 1.

**Table 3:** Analyses of indirect effects of model.

Variable relationships	Indirect effect	Lower	Upper
Indirect Effect			
Good personality → Orientation to meaning → Social well-being	0.24^***^	0.20	0.29
Good personality → Orientation to pleasure → Social well-being	0.00	−0.01	0.003
Total Indirect Effect	0.24^***^	0.20	0.28
Direct Effect	0.41^***^	0.33	0.48
Total Effect	0.65^***^	0.59	0.71

^***^p < 0.001.

## 4. Discussion

This study aimed to examine the association between good personality and social well-being, and the mediating role of orientations to meaning and pleasure. As it was hypothesized, a positive relationship between good personality and social well-being was demonstrated, which was mediated by meaning orientation but not pleasure orientation. This supported Lent’s normative model of well-being (18) and the cognition-instrumental model of well-being (41).

The finding that good personality and social well-being correlate positively was consistent with previous research (9, 12–15). These studies found an association between big five personality and social well-being in different cultures (37). There is also indirect evidence from a longitudinal study conducted in Chinese showing that Junzi personality (i.e., ideal persons) predicted interpersonal competence and satisfaction, which is an important component of social well-being (67). The positive relationship found here coincides with the Confucian cultural view, which strongly emphasizes virtue as a prerequisite for pleasure and holds that “Doing good deeds leads to happiness. Good deeds are the foundation of happiness. One cannot achieve happiness if s/he lacks goodness. The pursuit of happiness always enhances one’s goodness, and the maximum satisfaction is attained when goodness is perfected.” One of the theoretical contributions of this study is its combination of traditional philosophical, theoretical frameworks and culturally relevant personality notions to describe personal change and provide insights into social well-being in the context of Chinese culture.

Another major theoretical contribution of this study is that the results support Lent’s normative model of well-being (18) and the cognitive instrumental model proposed by Tkach and colleagues (41). The main finding of a mediating role of meaning orientation rather than pleasure orientation between good personality and social well-being indicates that good personality can facilitate social well-being by assigning individual talents in doing things that benefit humanity instead of entertaining oneself (68). This might be because orientation to meaning affects one’s emotional regulation (56, 62) and attention distribution (e.g., to their surroundings) (63), resulting in more resources available for configuring well-being. By contrast, orientations to pleasure did not lead to long-term but only momentary emotional enhancements (62).

The practical implication of this research includes improving Chinese citizen’ social well-being through cognitive intervention (e.g., orientation to meaning). The results may also inspire individuals to be kind. One’s sense of social well-being can be increased by acting morally, raising one’s sense of purpose and, ultimately, willingness to act morally. Furthermore, the present findings may provide some guidance in character cultivation and moral education.

Several limitations exist in this study. First, although all questionnaires used have good psychometric characteristics, they are self-reported measures. In addition, given the cross-sectional nature of this study, the causal relationship between the variables cannot be identified. To establish their causal relationship, longitudinal studies or laboratory experiments are needed in the future. Last, research involving more diverse ethnic and racial samples helps examine the generalization of the present findings.

## 5. Conclusion

According to the results of the current study, having good personality was positively associated with social well-being. Importantly, orientation to meaning partially mediate their relationship, which supported the normative and cognition instrumental models of well-being. Longitudinal research is called for in the future to clarify their causal relationship.

## Data availability statement

The original contributions presented in the study are included in the article/supplementary material, further inquiries can be directed to the corresponding author.

## Ethics statement

Ethics approval for this study was obtained from Beijing Normal University (IRB Number: 202208220094). Written informed consent to participate in this study was provided by the participants’ legal guardian/next of kin.

## Author contributions

XX: conceptualization, methodology, software, formal analysis, investigation, and writing—original draft preparation. YaL: conceptualization, validation, review, and editing. LJ: validation, data curation, review, and editing. YW: resources and data curation. MY: data curation and writing—review and editing. YiL: review and editing. YZ: resources and data curation. YX: funding acquisition and supervision. All authors contributed to the article and approved the submitted version.

## Funding

There are two funding sources for this study: the National Natural Science Foundation of China (31671160) and the Major Project of the National Social Science Foundation (19ZDA363).

## Conflict of interest

The authors declare that the research was conducted in the absence of any commercial or financial relationships that could be construed as a potential conflict of interest.

## Publisher’s note

All claims expressed in this article are solely those of the authors and do not necessarily represent those of their affiliated organizations, or those of the publisher, the editors and the reviewers. Any product that may be evaluated in this article, or claim that may be made by its manufacturer, is not guaranteed or endorsed by the publisher.
